# Effects of Microalgae-Based Nutraceuticals on Muscle Composition and Intestinal Function in Juvenile Gilthead Seabream Fed Plant Protein-Based Diets

**DOI:** 10.3390/ani16091350

**Published:** 2026-04-28

**Authors:** Alba Galafat, Isabel del Carmen Ruiz-Rodríguez, Alejandro Morcillo-Guillén, Anyell Caderno, Milagrosa Oliva, María Isabel Sáez, Antonio Jesús Vizcaíno, Tomás F. Martínez, Juan Antonio Martos-Sitcha, Francisco Javier Alarcón-López

**Affiliations:** 1 Departamento de Biología y Geología, Escuela Superior de Ingeniería, Universidad de Almería, La Cañada de San Urbano, 04120 Almería, Spain; irr646@ual.es (I.d.C.R.-R.); amg738@ual.es (A.M.-G.); msc880@ual.es (M.I.S.); avt552@ual.es (A.J.V.); tomas@ual.es (T.F.M.); falarcon@ual.es (F.J.A.-L.); 2 Departamento de Biología, Facultad de Ciencias Marinas y Medioambientales, Instituto Universitario de Investigación Marina (INMAR), Campus de Excelencia Internacional del Mar (CEI·MAR), Universidad de Cádiz, 11519 Puerto Real, Spain; anyell.caderno@uca.es (A.C.); milagrosa.oliva@uca.es (M.O.); juanantonio.sitcha@uca.es (J.A.M.-S.); 3 LifeBioencapsulation S.L., Parque Científico PITA, El Alquián, 04131 Almería, Spain

**Keywords:** plant-based diets, dietary fortification, growth performance, proximate composition, oxidative status, intestinal functionality, nutraceuticals, microalgae

## Abstract

This study evaluated the effects of dietary fortification of high plant protein aquafeeds with two microalgae-based functional ingredients on growth, muscle composition, oxidative status, digestive function, and intestinal morphology in juvenile *Sparus aurata* with an average body weight of 28.4 g. Four diets were tested: a control with high fishmeal and fish oil (CTF), a plant-based diet containing 5% fishmeal and 5% fish oil (CTV), and two CTV diets supplemented with 1% LB-IMMUNOboost (IB10) or 1% LB-LIVERprotect (LP10). Fish fed CTV and LP10 showed reduced growth compared to CTF, while IB10 partially mitigated these effects. High plant dietary inclusion reduced muscle protein and increased lipid content, except in IB10-fed fish. Reduction in fishmeal and fish oil decreased muscle saturated fatty acids, EPA, and DHA. Despite similar HUFA levels in the plant-based diets, IB10 and LP10 significantly reduced lipid peroxidation, indicating a direct antioxidant effect of the functional ingredients. Digestive enzyme activities were impaired in CTV-fed fish but partially recovered in IB10 and LP10, particularly IB10. Histology revealed shorter intestinal folds and more goblet cells in high plant protein diets, especially LP10, potentially impairing absorption. Overall, microalgae-based functional ingredients, particularly LB-IMMUNOboost, partially alleviated these adverse effects, supporting their use in more sustainable aquafeed formulations.

## Simple Summary

This study tested how adding two microalgae-based ingredients to plant-based diets affects juvenile *Sparus aurata*. Four diets were compared: a fishmeal/fish oil control, a plant-based diet, and the plant-based diet supplemented with either LB-IMMUNOboost or LB-LIVERprotect. The growth performance of fish fed with the plant-based diet was negatively affected, and specimens had lower protein and increased lipid contents in muscle. LB-IMMUNOboost helped to improve growth, muscle protein, antioxidant status, and digestive enzymatic activity. Histology showed that high plant content-based diets shortened intestinal folds and increased goblet cells, which might reduce intestinal absorption. Overall, adding microalgae-based nutraceuticals, especially LB-IMMUNOboost, helped to partially mitigate the negative effects of plant-based diets and could support more sustainable aquafeeds.

## 1. Introduction

Aquaculture faces significant challenges regarding the sustainable use of resources, particularly in relation to fishmeal, an ingredient traditionally employed for feeding farmed fish due to its nutritional quality [1,2,3]. However, the growing global demand for aquaculture products, aimed at meeting the elevated protein requirements of an expanding population, has intensified pressure on fishery resources used for fishmeal production. This has led to the worrying overexploitation of fishing grounds and a decline in the availability of key species [2,3,4]. This situation highlights the urgent need to identify more sustainable and efficient alternatives capable of fulfilling the nutritional needs of aquaculture fish.

In this regard, plant-based protein ingredients represent an alternative to fishmeal, owing to their adequate availability and economic viability [2,5,6]. Moreover, plant-based feedstuffs exhibit a nutrient composition well-suited for aquafeed formulation [5,7]. Numerous studies have explored the replacement of fishmeal in feeds with a variety of plant proteins at different inclusion levels and across various fish species, demonstrating positive outcomes in terms of growth, feed efficiency, and muscle proximate composition [5,6]. However, the presence of antinutritional compounds, which can compromise digestive health and the overall condition of farmed organisms, represents a limitation for the inclusion of high dietary levels of terrestrial plant-based feedstuffs, especially for feeding carnivorous species, such as gilthead seabream [5,8,9]. Additionally, the high fiber content, an unbalanced amino acid profile, and low palatability are also unfavorable characteristics of these raw materials, which restrict their widespread utilization in aquafeeds [5,10].

These limitations have driven a growing interest in the use of functional ingredients in feed formulation, which gained importance recently and emerged as a novel trend in the sector due to its potential to enhance the health and performance of aquaculture organisms [7,11,12]. Dietary fortification offers a range of benefits for animals, such as improvements in growth, nutrient digestibility, digestive function, immune status, and disease resistance [13,14], as well as in the quality of the final product, potentially improving the composition of the filet or the color of farmed aquatic organisms [14]. From probiotics and prebiotics promoting a healthy gut microbiome to enzymes improving nutrient digestibility, these substances are designed to optimize performance and efficiency in aquaculture production systems [15]. Indeed, this aspect has become a key component of preventive strategies implemented in fish farms [16].

In this sense, microalgae have emerged as a potential functional ingredient in aquaculture feeds, garnering increasing attention as a raw material [17,18]. Microalgae are a rich source of essential nutrients, including long-chain omega-3 fatty acids, high-quality protein, vitamins, and minerals, which can enhance the health and performance of cultured aquatic organisms [15,19]. Additionally, microalgae contain a wide range of bioactive compounds, such as pigments with antioxidant properties, which not only improve the coloration of fish and shrimp, thereby increasing their commercial appeal, but also provide antioxidant capacity, making them highly valuable [14,19,20].

Among the most notable effects of dietary fortification with microalgae-based products are improvements in digestive processes and nutrient absorption, as well as the restoration of intestinal health [21]. In this context, special attention has been given in recent years to the intestinal health in fish, as a healthy gut is closely linked to proper digestive functionality and the effective absorption and assimilation of nutrients [21]. This aspect is particularly significant when using feeds formulated with high content of plant protein ingredients, as these functional components can counteract the adverse effects associated with such diets [22].

LB-IMMUNOboost and LB-LIVERprotect (LifeBioencapsulation S.L.) are functional ingredients derived from microalgae, designed to exert distinct physiological effects. LB-IMMUNOboost is intended to support immune function during early developmental stages, modulating cellular and humoral defense responses and enhancing resistance to disease and environmental stressors. In contrast, LB-LIVERprotect is formulated to support hepatic function, promote the synthesis of phospholipids, and act as a lipotropic agent potentially involved in the prevention of excessive lipid accumulation in the liver. A previous study has evaluated the effects of these two microalgae-derived feed ingredients on metabolism, hepatic, intestinal electrophysiology, intestinal histology, and stress regulation in juvenile gilthead seabream fed diets with a high inclusion of plant-based raw materials [23]. Nevertheless, there is still limited information regarding how these ingredients influence muscle composition, lipid oxidation, and digestive functionality when plant protein ingredients constitute the main dietary protein source. On this basis, it was hypothesized that the dietary inclusion of LB-IMMUNOboost and LB-LIVERprotect would mitigate the adverse effects of plant-based diets in juvenile gilthead seabream, improving muscle proximate and fatty acid composition, enhancing digestive functionality, and reducing lipid oxidation.

Therefore, the objective of the present study was to evaluate the potential of these two microalgae-derived nutraceutical ingredients to counteract the negative effects associated with plant-based diets on growth, proximate composition, fatty acid profile, muscle lipid oxidation, and digestive functionality in juvenile gilthead seabream (*Sparus aurata*).

## 2. Materials and Methods

### 2.1. Experimental Diets

Four isonitrogenous (43% dry weight) and isolipidic (17% dry weight) experimental diets were formulated at the CEI·MAR-University of Almeria facilities (Unidad de Piensos Experimentales, Almeria, Spain) as follows: CTF diet, with an ingredient composition mimicking commercial feeds currently available for *S. aurata* (including 20% fishmeal and 9% fish oil), and without functional ingredients, CTV diet, a diet formulated with only 5% FM and 5% FO with high content of vegetal proteins and oils, and the supplementation of the CTV diet with a 1% (10 g per kg of feed) of LB-IMMUNOboost (IB10) or LB-LIVERprotect (LP10) nutraceuticals provided by LifeBioencapsulation S.L. (Almería, Spain; https://lifebioencapsulation.com/index.php/productos/ (accessed on 10 April 2026)). Thus, LB-IMMUNOboost is a concentrated product composed of 500 g kg^−1^ of enzymatically hydrolyzed yeast cell wall and 500 g kg^−1^ of a hydrolyzed microalgae blend (6:4) containing *Chlorella* sp., and *Microchloropsis* sp. LB-LIVERprotect is a concentrated product based on 700 g kg^−1^ of a blend (3:3:4) of artichoke powder (from *Cynara scolymus* leaves containing 25,000 ppm total polyphenols kg^−1^), freeze-dried marc grape polyphenolic extract (containing 37,500 ppm total polyphenols kg^−1^), and choline salts + 300 g kg^−1^ of a hydrolyzed microalgae blend (8:2) composed of *Arthrospira* sp. and *Microchloropsis* sp. For performing the enzymatic hydrolysis of yeast and each type of microalgal blend contained in both nutraceuticals, the biomasses were separately suspended (120 g dry weight L^−1^) in 50 mM sodium citrate buffer (pH 5.0) and incubated at 50 °C under agitation for 5 h with different carbohydrases (xylanase: 20,000 U g^−1^ and glucanase; 30,000 U g^−1^), cellulase (10,000 U g^−1^), and protease (10,000 U g^−1^) at an enzyme-to-substrate ([E]/[S]) ratio of 0.05. After that, the reaction mixture was heated for 15 min at 80 °C in order to inactivate the hydrolytic enzymes. Hydrolysates were dried using a freeze-drier (Telstar LyoQuest 55 PLUS, Telstar, Terrassa, Spain) and then the rest of the compounds were incorporated to constitute each one of the functional ingredients tested. The formulation and chemical composition of the different experimental diets are shown in Table 1 and Table 2.

### 2.2. Feeding Trial and Sampling

The feeding trial was carried out at the indoor facilities of the Aquaculture Technological Centre (CTAQUA, El Puerto de Santa María, Cádiz, Spain; Operational Code REGA ES110270000411) as described by Caderno et al. [23]. In short, 360 gilthead seabream juveniles (*Sparus aurata*) were previously acclimated to this facility for a period of 14 days. Then, fish with an average weight of 28.4 ± 0.2 g, were randomly distributed in 12 tanks with 400 L capacity and adjusted to a final volume of 350 L (2.50 ± 0.03 kg m^−3^ initial stocking density) coupled to a recirculating aquaculture system (RAS) equipped with both physical and biological filters. During the feeding trial, the experimental diets were supplied ad libitum manually, twice a day, 6 days per week, for 90 days. Each diet was administered to triplicate tanks. The number of replicate tanks per treatment was established considering standard experimental practice in aquaculture trials and supported by a priori sample size/power estimation using a statistical calculation tool (GRANMO), taking into account expected variability and experimental sensitivity. The feeding trial was carried out blindly, in such a way that the aquafeeds were labeled with four colors but with no reference to their composition, eliminating any source of subjectivity. The oxygen content of outlet water remained higher than 85% saturation, temperature (21–22 °C), 36 ‰ water salinity, and a photoperiod followed the natural changes at our latitude (36°35′06″ N; 06°13′48″ W).

At the end of the feeding trial (90 days), fish were fasted for 24 h prior to a final sampling, including biometric parameters. Specimens were transferred to a new tank containing 2-phenoxyethanol (1 mL L^−1^ seawater) to overdose for euthanasia. Whole intestines (*n* = 12) were extracted for the determination of digestive enzymes (four specimens per replicate tank). Moreover, samples of proximal intestine from three fish per tank (*n* = 9) were obtained for analysis of histology and ultrastructure of mucosa, through light microscopy and scanning (SEM) electron microscopy. In addition, fish muscle samples (*n* = 9) were taken for proximal composition and fatty acids profile analysis, as well as analysis of lipid oxidation.

Animals were handled and kept following the guidelines for experimental procedures in animal research of the Ethics and Animal Welfare Committee of the University of Cadiz, according to European Union (2010/63/UE) and Spanish (RD 53/2013 and RD 118/2021) regulations. The Ethical Committee from the Andalusian Government approved the feeding trial (# 23/10/2019/176).

### 2.3. Proximate Composition and Fatty Acid Profile of Feeds and Muscle

The chemical composition of the diets and fish muscle (*n* = 9) was determined following the AOAC [24] procedures for dry matter and ash. Crude protein (N × 6.25) was determined by elemental analysis (C: H: N) with a Fisons CHNS/O EA 1108 analyzer (Fisons Carlo Erba Instruments, Lakewood, NJ, USA). The lipid content was determined following the procedure described by Folch et al. [25]. Fatty acid (FA) profiles of feeds and muscle samples (*n* = 9) were determined by gas chromatography (Hewlett Packard, 4890 Serie II, Hewlett Packard Company, Avondale, PA, USA) following the method described by Rodríguez-Ruiz et al. [26], using a modification of the transesterification method described by Lepage and Roy [27] that requires no prior separation of the lipid fraction.

### 2.4. Muscle Lipid Peroxidation

Lipid oxidation in muscle samples (*n* = 9) was assessed by quantifying thiobarbituric acid-reactive substances (TBARS). The procedure followed Buege and Aust [28]. Briefly, 1 g of muscle tissue was homogenized in 4 mL of a solution containing 5 mM NaH_2_PO_4_ and 0.1% (*v*/*v*) Triton X-100 (Sigma-Aldrich, St. Louis, MO, USA). The homogenate was then centrifuged at 10,000× *g* for 20 min at 4 °C. After centrifugation, supernatants were collected and mixed (1:5, *v*/*v*) with a 2-thiobarbituric acid (TBA, T5500, Merck, Madrid, Spain) reagent consisting of 0.375% (*w*/*v*) TBA, 15% (*w*/*v*) TCA, 0.01% (*w*/*v*) 2,6-di-tert-butyl-4-methylphenol (BHT, B1378, Merck, Madrid, Spain) and 0.25 N HCl. This mixture was incubated by heating for 15 min and subsequently centrifuged at 3600× *g* for 10 min at 4 °C. Finally, the absorbance of the resulting supernatants was recorded at 535 nm. TBARS levels were expressed as mg of malondialdehyde (MDA, 63287, Merck, Madrid, Spain) per kg of muscle tissue, using a calibration curve prepared with MDA standards.

### 2.5. Intestinal Enzymatic Activities

Intestinal samples were processed individually to obtain crude extracts for enzymatic activity measurements. Tissue samples were homogenized manually in distilled water at 4 °C to reach a final concentration of 0.5 g mL^−1^. The resulting homogenates were centrifuged at 13,000× *g* for 12 min at 4 °C, and the supernatants were collected and stored at −20 °C until analysis. Trypsin and chymotrypsin activities were determined using 0.5 mM BAPNA (Na-benzoyl-DL-arginine-4-p-nitroanilide, B4875, Merck, Madrid, Spain) and 0.2 mM SAPNA (N-succinyl-(Ala)_2_-Pro-Phe-p-nitroanilide, S7388, Merck, Madrid, Spain), respectively. Assays were performed in 50 mM Tris-HCl buffer containing 10 mM CaCl_2_ at pH 8.5, following the methods of Erlanger et al. [29] and Del Mar et al. [30]. Enzyme activity was expressed in units (U), defined as the amount of enzyme releasing 1 μmol of p-nitroanilide (pNA) per minute, quantified spectrophotometrically at 405 nm using an extinction coefficient of 8800 M cm^−1^. Total alkaline protease activity was measured according to Alarcón et al. [31], using casein (5 g L^−1^, E0789, Merck, Madrid, Spain) as substrate in 50 mM Tris-HCl buffer (pH 9.0). One unit of activity was defined as the enzyme amount releasing 1 μg of tyrosine per minute in the reaction mixture, based on an extinction coefficient of 0.008 μg^−1^ mL^−1^ cm^−1^, with absorbance read at 280 nm. Leucine aminopeptidase activity was assayed using 2 mM L-leucine-p-nitroanilide (LpNa, L9125, Merck, Madrid, Spain) in 100 mM Tris-HCl buffer (pH 8.8), following Pfleiderer [32]. Alkaline phosphatase activity was determined according to Bergmeyer [33], using p-nitrophenyl phosphate (487663, Merck, Madrid, Spain) in a buffer containing 1 M diethanolamine and 1 M MgCl_2_ at pH 9.5.

### 2.6. Histological Analysis of the Intestinal Mucosa

Intestinal samples were fixed for 24 h in phosphate-buffered formalin (4% *v*/*v*, pH 7.2), followed by dehydration and paraffin embedding using standard histological procedures. Proximal intestinal segments were oriented to obtain 5 μm transversal sections along the gut lumen axis. All sections were stained with Periodic acid–Schiff (PAS). Histological examination was performed using a light microscope (OLYMPUS IX51, Olympus, Madrid, Spain) equipped with a CC12 digital camera (Olympus Soft Imaging Solutions GmbH, Münster, Germany). Image acquisition and analysis were conducted with ImageJ software (version 1.45; National Institutes of Health, Bethesda, MD, USA). Morphometric parameters included fold length, enterocyte height, lamina propria thickness, and goblet cell density per 100 µm of mucosal fold. For each animal (*n* = 9 per dietary treatment), 10 independent measurements were taken in both proximal and distal intestinal regions to evaluate potential modifications in mucosal architecture, as previously described in fish fed diets containing plant protein sources [34]. For scanning electron microscopy (SEM), additional intestinal samples were fixed under the same conditions (4% formaldehyde in phosphate buffer, pH 7.2) for 24 h. After fixation, tissues were washed, dehydrated in ethanol, and subjected to critical point drying using absolute ethanol as intermediate fluid and CO_2_ as transition fluid (critical point dryer CDP 030, Leica Microsystems, Barcelona, Spain). Dried samples were mounted on stubs and coated with graphite (PELCO Colloidal Graphite, Ted Pella Inc., Redding, CA, USA) and gold (SCD 005 Sputter Coater, Leica Microsystems, Barcelona, Spain). SEM observation was carried out using a HITACHI S-3500 scanning electron microscope (Hitachi High-Technologies Corporation, Tokyo, Japan). Image analysis was performed with UTHSCSA ImageTool software 3.0 (University of Texas Health Sciences Center, San Antonio, TX, USA), and the enterocyte apical area (EA) was quantified.

### 2.7. Statistical Analysis

All dietary treatments were tested in triplicate tanks, and the analysis of biological samples was conducted with a minimum of three repetitions. The tank was considered the experimental unit. For measurements with multiple observations per individual [chemical composition and fatty acid profile (*n* = 9), digestive enzymatic activities (*n* = 12), lipid oxidation (*n* = 9), histological and ultrastructural study (*n* = 9)], values were averaged by tank to maintain independence of experimental units and avoid pseudoreplication. Outliers were identified using the Tukey method (1.5 × IQR) and removed from subsequent analyses. Data were expressed as mean ± SEM. All variables measured were continuous. Comparison of means was carried out by one-way ANOVA with a significant level set at 5% (*p* < 0.05) for variables meeting assumptions of normality (Shapiro–Wilk test) and homoscedasticity (Levene test), followed by Tukey’s HSD for multiple comparisons between treatments. When the data did not meet the ANOVA assumptions, a Kruskal–Wallis one-way analysis of variance on ranks was used, and results were presented with Box-and-Whisker Plots. Data expressed as percentage (%) were arcsine (×1/2)-transformed. All statistical analyses were performed with Statgraphics Plus 19.0 (Warrenton, VA, USA) software.

## 3. Results

### 3.1. Zootechnical Parameters and Chemical Composition Analysis

#### 3.1.1. Growth Performance

**Figure 1:**
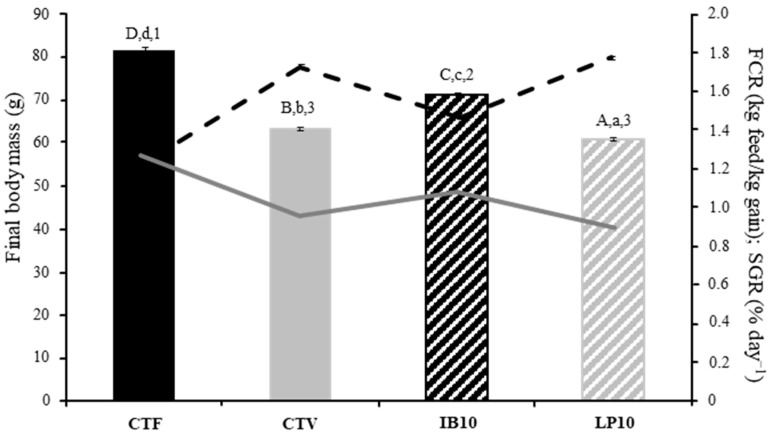
Growth performance (bars), specific growth rate, SGR (solid line) and feed conversion ratio, and FCR (dashed line) parameters of gilthead seabream juveniles fed with experimental diets. CTF: fishmeal-based control diet containing 20% fishmeal and 9% fish oil; CTV: plant-based control diet containing 5% fishmeal and 5% fish oil; IB10: CTV supplemented 1% LB-IMMUNOboost; LP10: CTV supplemented 1%. Values are mean ± SEM (*n* = 3). Uppercase letters indicate significant differences in the final body mass of fish fed with different dietary treatments. Lowercase letters indicate significant differences in SGR among dietary treatments. Numbers indicate significant differences in FCR among dietary treatments (*p* < 0.05).

The final weight differed among experimental groups, being higher for the control batch (CTF) followed by IB10, presenting specimens fed CTV and LP10 diets the lowest values (Figure 1). SGR showed a similar trend, with the highest values observed in fish from CTF dietary treatment, followed by IB10, while CTV and LP10 had the lowest values. FCR was highest in the CTV and LP10 groups, followed by IB10, whereas CTF exhibited the lowest values for this parameter.

#### 3.1.2. Proximate Composition

Table 3 shows muscle proximal composition in gilthead seabream juveniles fed with the different experimental diets for 90 days. Overall, a decrease in muscle protein content was observed in fish fed the CTV and LP10 diets, compared to fish in the CTF group. However, fish fed with the diet IB10 did not differ from the control group (CTF). Muscle lipid content significantly increased in all the fish fed the diets with high content of plant protein ingredients (CTV, IB10 and LP10) compared to fish from the CTF group. Furthermore, fish fed CTV and LP10 showed the highest values. Ash and moisture did not differ among dietary treatments.

#### 3.1.3. Fatty Acid Profile

The muscle fatty acid profile of juvenile gilthead seabream fed the different experimental diets is detailed in Table 4. The CTV group showed the lowest content of SFA in muscle compared to the rest of the dietary groups. Dietary fortification with the functional ingredients significantly increased these fatty acids in muscle but without reaching the value observed in the CTF group. No differences were obtained in MUFA and PUFA content. CTV, IB10 and LP10 significantly increased 18:1n9 and 18:2n6 content and decreased ARA, EPA and DHA content in muscle, compared to CTF. The retention of these fatty acids was not affected by the inclusion of the microalgae-based ingredients relative to CTV. Additionally, the n-3/n-6 ratio was higher in the CTF group compared to the other treatments, whereas no relevant differences were observed between the groups receiving the functional ingredients and CTV.

#### 3.1.4. Muscle Lipid Oxidation

**Figure 2:**
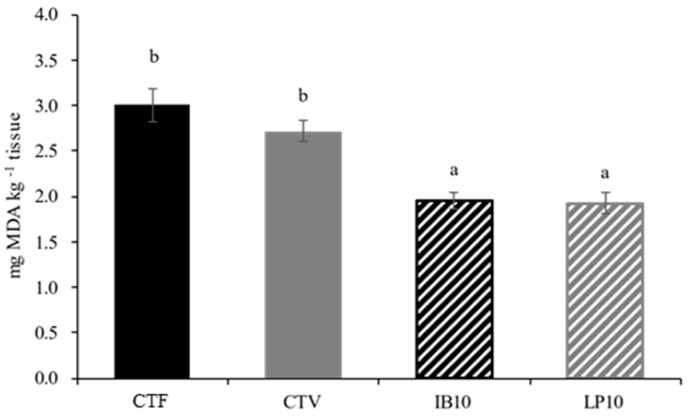
Muscle lipid oxidation, estimated as TBARS content (mg MDA kg^−1^ tissue) in *S. aurata* juveniles fed the diets. CTF: fishmeal-based control diet containing 20% fishmeal and 9% fish oil; CTV: plant-based control diet containing 5% fishmeal and 5% fish oil; IB10: CTV supplemented 1% LB-IMMUNOboost; LP10: CTV supplemented 1% LB-LIVERprotect. Values are mean ± SEM (*n* = 9). Bars with different lowercase letters indicate significant differences (*p <* 0.05).

Figure 2 shows the TBARS content in the muscle of juvenile gilthead seabream fed with experimental diets. A noticeable decrease in values was evidenced in fish fed IB10 and LP10 diets compared to the CTF and CTV groups.

### 3.2. Digestive Functionality

#### 3.2.1. Analysis of Intestinal Enzymes

**Figure 3:**
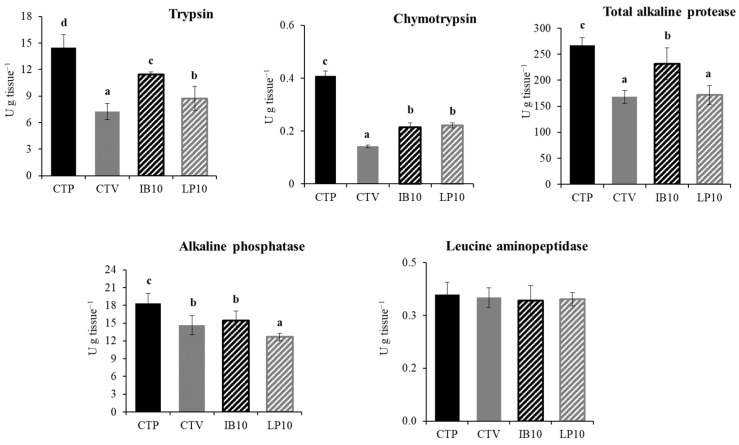
Digestive enzyme activities (U g^−1^ tissue) were determined in the intestinal extract of gilthead seabream fed the diets. CTF: fishmeal-based control diet containing 20% fishmeal and 9% fish oil; CTV: plant-based control diet containing 5% fishmeal and 5% fish oil; IB10: CTV supplemented 1% LB-IMMUNOboost; LP10: CTV supplemented 1% LB-LIVERprotect. Values are mean ± SEM (*n* = 12). Different lowercase letters indicate significant differences among treatments (*p* < 0.05).

Figure 3 shows the intestinal enzyme activities in gilthead seabream fed the experimental diets. The CTV diet reduced the activities of trypsin, chymotrypsin, total alkaline protease, and alkaline phosphatase compared to fish fed the CTF diet. Dietary fortification with the functional ingredients significantly increased trypsin and chymotrypsin activities, reaching approximately 58% and 51% higher than CTV in the IB10 group, and 21% and 56% higher than CTV in the LP10 group, respectively. The activity of total alkaline protease increased significantly only in the IB10 group (approximately 38% relative to CTV), whereas no significant changes were observed in LP10. By contrast, alkaline phosphatase activity remained reduced in the CTV, IB10, and LP10 groups compared with CTF, with LP10 showing significantly lower activity than the other treatments. Leucine aminopeptidase activity did not differ significantly among experimental groups.

#### 3.2.2. Structural Analysis of the Intestinal Mucosa

**Figure 4:**
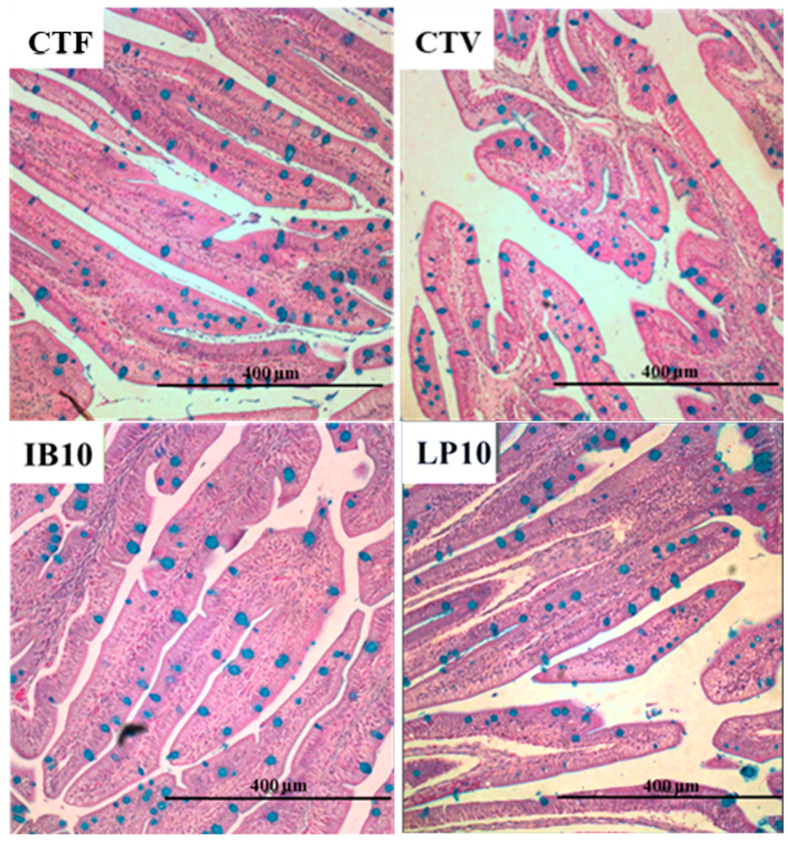
Photomicrographs of the anterior intestine in *S. aurata* juveniles fed the experimental diets (PAS staining). CTF: fishmeal-based control diet containing 20% fishmeal and 9% fish oil; CTV: plant-based control diet containing 5% fishmeal and 5% fish oil; IB10: CTV supplemented 1% LB-IMMUNOboost; LP10: CTV supplemented 1% LB-LIVERprotect.

**Figure A1:**
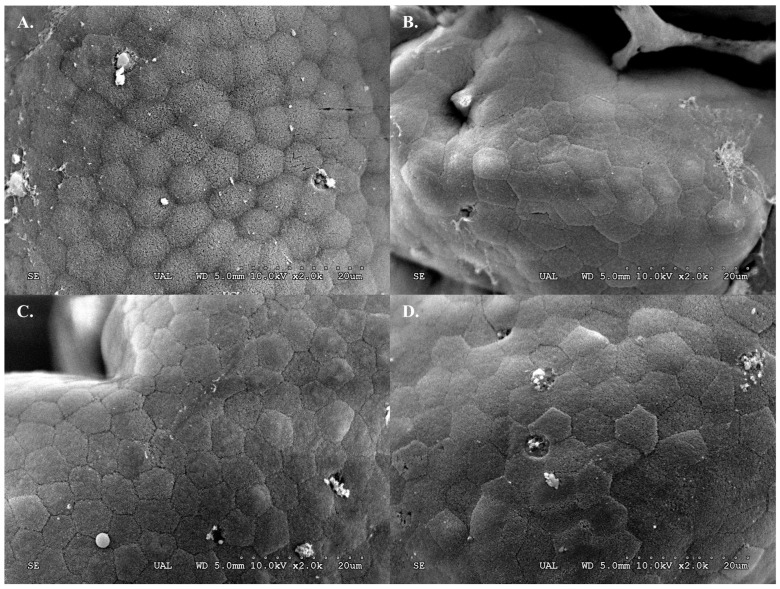
SEM micrographs of the apical mucosa surface of anterior intestine in gilthead seabream juveniles fed the different experimental diets. (**A**) CTF; (**B**) CTV; (**C**) IB10; (**D**) LP10.

Figure 4 shows a detail of the intestinal sections of fish fed the experimental diets. The results obtained from the histomorphometry analysis of the intestinal mucosa are detailed in Table 5. Fish fed CTF presented longer intestinal folds (FL) compared with the rest of the specimens fed the other treatments, but only significant differences were found with respect to LP10. Total enterocyte height (TEH) was lower in CTV, but IB10 and LP10 did not differ with respect to CTF. Lamina propria thickness (LPT) tended to increase in CTV, IB10 and LP10 groups, but only fish fed LP10 significantly differed from the CTF group. Finally, the number of goblet cells was similar in specimens fed with the CTF, CTV and LP10 diets, but the count significantly lowered in the IB10 group. SEM images evidenced a normal appearance in the surface of the intestinal mucosa in all dietary treatments (Figure A1) without a significant difference in the apical area of the enterocytes.

## 4. Discussion

Aquaculture has established itself as one of the leading agri-food activities worldwide, playing a key role in supplying food for a continuously growing population [15,35]. However, the dependence on fishmeal for feed production remains one of the major challenges facing the sector [5,36]. Among the most extensively studied alternatives, plant-based proteins have emerged in recent years as a viable option for replacing conventional ingredients in aquaculture diets [37,38,39]. Nevertheless, high inclusion levels of plant-based meals may negatively affect growth, digestive functionality, and overall fish welfare [40,41]. To overcome these limitations, complementary nutritional strategies have been explored to mitigate such adverse effects and enhance the utilization of these raw materials [42,43,44]. Within this framework, the present study evaluated the potential of two algae-based functional ingredients to assess their potential influence on responses in fish fed with diets containing high levels of plant-based ingredients.

The results obtained showed a reduction in growth performance in fish fed a plant-based diet (CTV), which may be associated with the negative effects derived from the presence of antinutritional factors in these raw materials [5,45,46,47,48]. The diet supplemented with LB-IMMUNOboost exhibited a partial improvement in growth performance compared to the CTV diet, although fish did not reach the final values observed in the CTF group, in agreement with previous studies reporting increased growth in fish fed diets enriched with microalgae and hydrolyzed yeast [17,18,49,50]. Such effects have been attributed to the nutraceutical properties of microalgae, which may enhance growth through increased protein and triglyceride deposition in muscle tissue [51]. In contrast, fish fed the LP10-supplemented diet presented the lowest growth values among all treatments. These variations in growth likely reflect the action of the functional ingredients and their physiological interactions rather than differences in the basic composition of the feeds. In the case of LP10, its composition, based primarily on plant extracts (artichoke and grape polyphenols) and microalgae, without the inclusion of yeast hydrolysate or other compounds that directly promote digestion and protein deposition [52], could explain the absence of a positive effect on growth, and proximate composition analysis of muscle showed a reduction in protein content in LP10 compared with CTF, which, despite the diets being formulated to be isoproteic and isolipidic, reinforces the notion that LP10 did not promote protein deposition in muscle.

Fish fed the CTV diet showed a reduction in muscle protein content compared to the CTF group, likely associated with the high inclusion of plant proteins. This observation aligns with previous reports indicating that plant-based diets can induce modest decreases in muscle protein content relative to fishmeal-based diets [53]. Supplementation with LB-IMMUNOboost resulted in a slight increase in muscle protein relative to CTV, although differences were not statistically significant compared with either CTF or CTV. These findings are consistent with studies suggesting that the inclusion of yeast hydrolysates and microalgae in plant-based diets can improve protein digestibility and intestinal absorption, partially supporting muscle protein deposition [52,54]. Proteolytic digestive enzyme activities were also significantly enhanced in this group, suggesting a partial improvement in protein digestion that could increase substrate availability for muscle protein synthesis [55,56].

In terms of lipid content, fish fed plant protein-based diets showed an increase in muscle lipids compared to the fishmeal-based control (CTF), consistent with previous studies reporting that the replacement of fish oil with plant oils is associated with a higher muscle lipid fraction [57,58,59,60]. However, values observed in the IB10 experimental group were intermediate, which may indicate that part of the lipids were used as an energy source for muscle growth rather than being accumulated in the muscle, potentially reflecting the partial contribution of yeast hydrolysates and microalgae included in the diet, and possibly contributing to the higher weight gain observed in this group compared to CTV and LP10 [54,61].

The use of high proportions of plant meals in aquafeeds affects the fatty acid profile of fish muscle, an effect that is particularly pronounced in marine fish, whose capacity to synthesize long-chain PUFAs from their precursors is more limited [62]. Accordingly, the fatty acid profile of fish muscle in the present study varied depending on the diet provided. Specifically, SFA levels were lower in fish fed plant-based diets, which is consistent with the findings reported by Hussain et al. [5] in rainbow trout fed diets containing high proportions of plant ingredients. In particular, the deposition of myristic (14:0) and palmitic (16:0) acids in tissues was reduced in these fish, likely because these fatty acids serve as preferred substrates for β-oxidation, especially when diets are characterized by low n-3 HUFA levels [57,63]. Inclusion of functional ingredients in the IB10 and LP10 groups significantly increased SFA levels compared with the CTV group, although values did not reach those observed in CTF. Comparable results have also been reported in gilthead seabream fed diets containing *Schizochytrium* sp. and *Microchloropsis* sp. oils [64]. The relative recovery of SFA may have functional implications, as adequate levels of these fatty acids in muscle contribute to maintaining a more stable lipid-based energy supply, which is necessary to support active digestive processes and overall lipid homeostasis [65,66,67]. In this regard, fish in the IB10 and LP10 groups exhibited increased activities of trypsin, chymotrypsin, and total alkaline protease compared with CTV, indicating a potential enhancement in digestive efficiency and nutrient absorption capacity. In addition, histological evidence supports these observations: enterocyte height and intestinal fold length in IB10 and LP10 were closer to those observed in CTF, while lamina propria thickness and goblet cell counts showed adjustments consistent with a functionally active intestinal mucosa. Thus, the partial recovery of muscle SFA is accompanied by morphological and enzymatic indicators that suggest better preservation of intestinal functionality under plant-based diets.

On the other hand, and as expected, plant-based diets reduced EPA and DHA contents in the muscle of fish fed plant-based diets compared to those in the CTF group [5,63,64]. In this context, despite the high content of essential fatty acids reported for several microalgae species [64,68,69], the inclusion of algal-based functional ingredients in the present study was not sufficient to reverse the reduction in the muscle deposition of these fatty acids, which may be related to the low inclusion percentage (1%) used in the feed formulation.

Pigments in microalgae are secondary metabolites responsible not only for their coloration, but also for light energy absorption and protection against oxidative stress [20]. This latter function is particularly relevant for the development of new functional additives in aquaculture, since lipid peroxidation represents one of the major challenges during the cold storage of fish, leading to a reduction in shelf life and overall product quality [70]. In this regard, TBARS determination in muscle revealed a clear decrease in fish fed diets containing any of the evaluated functional ingredients when compared to the CTV and CTF experimental groups.

Although muscle lipid composition showed some variations between treatments (Table 4), these alone do not appear to explain the observed reduction in oxidation. Muscles from the treatments containing nutraceuticals exhibited a higher total lipid content, which, in principle, could increase susceptibility to peroxidation. Moreover, the total fraction of long-chain n-3 polyunsaturated fatty acids, which are most prone to oxidation, was similar across the plant-based diets. These findings suggest that, although lipid composition may exert some modulatory effect, the reduction in lipid peroxidation can be attributed predominantly to the direct antioxidant action of the compounds present in the nutraceuticals, including pigments and other natural antioxidants from microalgae and yeast extracts, which may neutralize reactive oxygen species and protect cellular membranes [20].

Numerous studies have assessed the effect of dietary microalgae inclusion on muscle oxidative status in different aquaculture species, reporting results that are consistent with those observed in the present study [43,71,72,73,74]. However, despite the presence of plant polyphenolic extracts in LB-LIVERprotect, this additional antioxidant input was not reflected in the muscle oxidative status of the fish, as values were similar to those of fish fed the IB10 diet.

Digestive enzymes play a crucial role in the digestion process, with alkaline proteases such as trypsin and chymotrypsin being primarily responsible for protein digestion in the intestine [73]. The activity of these enzymes can be modulated in response to the inclusion of different dietary ingredients [75]. In this context, the results obtained in the present study showed a reduction in the activity of digestive enzymes, including pancreatic enzymes such as trypsin, chymotrypsin, and total alkaline protease, associated with the use of plant-based diets. This negative effect is widely attributed to the presence of various anti-nutritional factors in plant meals, which can inhibit digestive proteases and reduce dietary protein digestibility [54,76]. However, particularly in fish fed the IB10 diet, a significant increase in the activity of trypsin, chymotrypsin, and total alkaline protease was observed when compared to the CTV treatment. Numerous studies have reported that dietary microalgae inclusion is associated with improvements in digestive functionality in different fish species [43,64,71,73,77,78]. Furthermore, the improvement observed in the IB10 dietary treatment may have been enhanced by the presence of yeast hydrolysates in LB-IMMUNOboost, which have also been associated with increased nutrient digestibility [79,80,81], potentially contributing to the improved growth performance observed in fish from this experimental group, compared to the other plant-based treatments [82].

On the other hand, the activity of the brush border enzymes in the intestinal mucosa is closely associated with nutrient assimilation processes, making it a useful marker of digestive efficiency [82]. In this regard, alkaline phosphatase activity was affected by changes in the dietary protein sources, with plant-based diets showing a significant reduction in enzyme activity. This effect may again be related to the presence of anti-nutritional factors in plant raw materials, which have been reported to impair the activity of these intestinal enzymes [54,75]. Moreover, these effects were more pronounced in fish fed the LP10 diet, which may be associated with a reduction in intestinal absorption processes. In addition, histological evaluation of the intestinal mucosa revealed a decrease in the length of the intestinal folds and an increase in the thickness of the lamina propria in this experimental group. These modifications may reflect morphological adaptation or tissue remodeling in response to the diet [83]. Although these changes were statistically significant, the overall epithelial morphology, including enterocyte height and goblet cell density, remained relatively stable, suggesting that there were no major alterations in intestinal architecture. In the IB10 group, a reduction in goblet cell number was observed, while fold length and enterocyte height remained similar to the CTF group. The decrease in goblet cells indicates a mucosal adjustment that could affect mucus secretion, with potential implications for the protection of the intestinal surface against irritants, pathogens, or luminal stress. Nevertheless, the overall intestinal structure is preserved, indicating that the mucosa maintains its basic architecture and absorptive capacity, while the changes primarily affect the specialized cellular composition of the epithelium. This finding reflects a moderate morphological response to the diet supplemented with the functional ingredient, with implications mainly related to mucosal barrier function and intestinal protection [84,85,86].

However, electrophysiological analysis of the intestine showed a general improvement in transepithelial electrical resistance (Rt) in fish fed the IB10 and LP10 diets, together with a reduction in apparent tissue permeability in the IB10 group compared to the CTV group, indicating an improvement in intestinal barrier function [23]. This functional recovery suggests that dietary supplementation with microalgae-based functional additives may partially counteract the negative effects of vegetable-based diets on epithelial integrity, even in the presence of histological alterations.

These results indicate that the lack of clear digestive effects of LB-LIVERprotect should not be interpreted as a negative impact on intestinal health or overall fish welfare. Rather, this functional additive is specifically designed to modulate metabolic homeostasis and hepatic function, enhancing energy storage capacity, maintaining liver structural integrity, and supporting the response to nutritional stress, particularly under extreme plant protein-based diet conditions. Previous studies have shown that supplementation with LB-LIVERprotect induces significant hepatic metabolic changes, including the attenuation of histological alterations and the regulation of metabolic stress markers, without directly affecting nutrient digestion or absorption [23]. Accordingly, the findings of the present study are consistent with this mode of action, as the additive, despite not eliciting increases in intestinal enzymatic activity or growth performance, contributes to preserving physiological integrity and metabolic efficiency, highlighting its value as a hepatic modulator in nutritional strategies involving high levels of marine ingredient replacement.

## 5. Conclusions

In conclusion, the experimental diets containing over 75% plant-based ingredients negatively affected growth performance, muscle composition, lipid quality, digestive enzyme activity, and intestinal functionality in fish, likely due to the presence of antinutritional factors that reduce protein digestibility and nutrient absorption. The inclusion of microalgae-derived functional ingredients partially counteracted these adverse effects. In particular, IB10 showed an intermediate performance between CTF and CTV for several indicators, with values between the two groups for growth, muscle composition, and digestive enzyme activity. LP10 had a more limited effect on growth and muscle, consistent with its formulation, which aimed to support liver function rather than directly enhance digestive physiology (see Caderno et al. [23]). These findings highlight the potential of microalgal-based additives as targeted nutritional tools, while emphasizing the importance of aligning functional ingredient selection with the intended physiological objectives. Further research is needed to optimize inclusion levels, elucidate the underlying mechanisms, and support the development of more efficient and sustainable plant-based feeds. Significantly, this study provides a unified experimental demonstration of how the functional design of microalgae-based additives can distinctly influence growth, metabolism, and digestive physiology in fish subjected to highly plant-based diets, offering new insights for feed formulation and aquaculture practices.

## Figures and Tables

**Table 1:** Ingredients and proximal composition (% dry matter) of the experimental diets.

Ingredients (g 100 g^−1^)	CTF	CTV	IB10	LP10
Fishmeal LT94 ^1^	20.00	5.00	5.00	5.00
Lysine ^2^	1.20	1.20	1.20	1.20
Methionine ^3^	0.50	0.60	0.60	0.60
LB-LIVERprotect ^4^				1.00
LB-IMMUNOboost ^5^			1.00	
Squid meal ^6^	2.00	2.00	2.00	2.00
Fishmeal hydrolysate CPSP90 ^7^	1.00	1.00	1.00	1.00
Krill meal ^8^	2.00	2.00	2.00	2.00
Wheat gluten ^9^	8.00	11.00	11.00	11.00
Soybean protein concentrate ^10^	26.00	36.00	36.00	36.00
Pea protein concentrate ^11^	7.50	10.60	10.60	10.60
Fish oil ^12^	9.00	5.00	5.00	5.00
Soybean oil ^13^	4.30	10.10	10.10	10.10
Soybean lecithin ^14^	1.00	1.00	1.00	1.00
Wheat meal ^15^	12.90	9.60	8.60	8.60
Monocalcium phosphate ^16^	0.50	0.80	0.80	0.80
Vitamin and mineral premix ^17^	2.00	2.00	2.00	2.00
Vitamin C ^18^	0.10	0.10	0.10	0.10
Guar gum ^19^	2.00	2.00	2.00	2.00
**Proximate composition (g 100 g^−1^)**				
Crude protein	43.10	43.10	42.90	42.80
Crude lipid	16.80	17.10	17.30	16.80
Ash	8.30	6.80	6.80	6.80
Fiber	4.6	5.4	5.7	5.3
Nitrogen-free extract ^20^	31.8	33.0	33.0	33.6
Gross energy (MJ kg^−1^ DM) ^21^	22.0	22.4	22.4	22.3

CTF: fishmeal-based control diet containing 20% fishmeal and 9% fish oil; CTV: plant-based control diet containing 5% fishmeal and 5% fish oil; IB10: CTV supplemented 1% LB-IMMUNOboost; LP10: CTV supplemented 1% LB-LIVERprotect. ^1^ With 69.4% crude protein, 12.3% crude lipid (Norsildemel, Bergen, Norway). ^2, 3^ Lorca Nutrición Animal SA (Murcia, Spain). ^4^ LB-LIVERprotect: (700 g/kg artichoke and marc grape polyphenolic extract and choline salts + 300 g/kg of a hydrolyzed microalgae blend composed by *Arthrospira* sp. and *Microchloropsis* sp.) (LifeBioencapsulation, Almería, Spain), 41% crude protein, 1% crude lipid, 24.5% ash, 13% moisture; 13,000 ppm total polyphenols kg^−1^. ^5^ LB-IMMUNOboost: (500 g/kg enzymatically hydrolyzed yeast cell wall + 500 g/kg of a hydrolyzed microalgae blend composed of *Chlorella* sp. and *Microchloropsis* sp.) (LifeBioencapsulation, Almería, Spain), 47% crude protein, 5.9% crude lipid, 13.7% ash, 7.5% moisture. ^6, 7, 8^ The 76%, 84% and 56% crude protein, respectively, were purchased from Bacarel (UK). CPSP90 is enzymatically pre-digested fishmeal. ^9^ The 78% crude protein (Lorca Nutrición Animal SA, Murcia, Spain). ^10^ Soycomil, 60% crude protein, 1.5% crude lipid (ADM, Poland). ^11^ Pea protein concentrate, 85% crude protein, 1.5% crude lipid (Emilio Peña SA, Spain). ^12^ AF117DHA (Afamsa, Spain). ^13^ Soybean oil (Aceites el Niño, Spain). ^14^ P700IP (Lecico, DE). ^15^ Local providers (Almería, Spain). ^16^ Andres Pintaluba, Spain. ^17^ *Lifebioencapsulation* SL (Almería, Spain). Vitamins (g kg^−1^): vitamin A (retinyl acetate), 2,000,000 UI; vitamin D3 (DL-cholecalciferol), 200,000 UI; vitamin E (Lutavit E50), 10 g; vitamin K3 (menadione sodium bisulphite), 2.5 g; vitamin B1 (thiamine hydrochloride), 3 g; vitamin B2 (riboflavin), 3 g; calcium pantothenate, 10 g; nicotinic acid, 20 g; vitamin B6 (pyridoxine hydrochloride), 2 g; vitamin B9 (folic acid), 1.5 g; vitamin B12 (cyanocobalamin), 0.01 g; vitamin H (biotin), 0.3 g; inositol, 50 g; betaine (Betafin S1), 50 g. Minerals (g kg^−1^): Co (cobalt carbonate), 0.065 g; Cu (cupric sulphate), 0.9 g; Fe (iron sulphate), 0.6 g; I (potassium iodide), 0.05 g; Mn (manganese oxide), 0.96 g; Se (sodium selenite), 0.001 g; Zn (zinc sulphate), 0.750 g; Ca (calcium carbonate), 18.6% (186 g); KCl, 2.41% (24.1 g); NaCl, 4.0% (40 g). ^18^ TECNOVIT, Spain. ^19^ Emilio Peña SA, Spain. ^20^ Calculated as 100 − (% crude protein + % crude lipid + % fiber + % ash). ^21^ Gross energy was estimated by energetic coefficients (kJ g^−1^): crude protein, 23.6; crude lipid, 38.9; Nfe, 16.7.

**Table 2:** Fatty acid profile (% of total fatty acids) of the diets.

	CTF	CTV	IB10	LP10
14:0	2.07	1.15	1.13	1.18
16:0	17.02	15.82	15.31	17.43
18:0	4.51	4.05	4.04	4.13
16:1n7	2.85	1.47	0.72	0.96
16:2n4	0.12	0.23	0.21	0.17
16:3n4	0.54	0.27	0.24	0.28
18:1n9	19.15	25.75	25.78	26.28
18:2n6	22.52	43.75	43.86	44.95
18:3n3	2.39	0.26	0.17	0.07
20:1n9	1.09	0.88	0.88	0.91
20:4n6, ARA	1.26	0.55	0.47	0.54
20: 4n3	1.70	0.27	0.13	0.39
20:5n3, EPA	5.51	2.62	2.49	2.67
22:5n3	0.81	0.40	0.40	0.41
22:6n3, DHA	12.88	7.02	6.86	7.18
Other fatty acids	4.97	2.27	2.10	2.07
ΣSFA	23.60	21.02	20.49	22.74
ΣMUFA	23.09	28.11	27.38	28.15
ΣPUFA	25.18	11.35	10.54	11.26
Σn3	23.92	12.84	10.07	10.72
Σn6	23.78	44.31	44.33	45.49
Σn9	1.09	0.88	0.88	0.91
n3/n6	1.01	0.30	0.23	0.24
EPA/DHA	0.43	0.37	0.36	0.37

CTF: fishmeal-based control diet containing 20% fishmeal and 9% fish oil; CTV: plant-based control diet containing 5% fishmeal and 5% fish oil; IB10: CTV supplemented 1% LB-IMMUNOboost; LP10: CTV supplemented 1% LB-LIVERprotect. ARA: Arachidonic acid; EPA: Eicosapentaenoic acid; DHA: Docosahexaenoic acid; SFA: saturated fatty acids; MUFA monounsaturated fatty acids; PUFA: polyunsaturated fatty acids.

**Table 3:** Muscle proximate composition (g 100 g^−1^ dry weight) and moisture (%) in *S. aurata* juveniles after 90 days of feeding the diets.

	CTF	CTV	IB10	LP10	*p*-Value
Total protein	69.34 ± 0.99 ^b^	61.94 ± 0.25 ^a^	65.38 ± 1.33 ^ab^	63.25 ± 0.10 ^a^	0.025
Total lipid	24.45 ± 0.40 ^a^	31.36 ± 0.31 ^c^	28.61 ± 0.18 ^b^	31.21 ± 0.45 ^c^	<0.001
Ash	6.10 ± 0.05	6.10 ± 0.04	6.21 ± 0.08	6.18 ± 0.04	0.557
Moisture	73.60 ± 1.05	74.07 ± 0.68	74.79 ± 0.42	74.88 ± 0.55	0.566

CTF: fishmeal-based control diet containing 20% fishmeal and 9% fish oil; CTV: plant-based control diet containing 5% fishmeal and 5% fish oil; IB10: CTV supplemented 1% LB-IMMUNOboost; LP10: CTV supplemented 1% LB-LIVERprotect. Values are mean ± SEM (*n* = 9). Values in the same row with different lowercase letters indicate significant differences among dietary treatments (*p* < 0.05).

**Table 4:** Muscle fatty acid profiles (% of total fatty acids) in *S. aurata* juveniles after 90 days of feeding the diets.

	CTF	CTV	IB10	LP10	*p*-Value
14:0	1.93 ± 0.01 ^b^	1.44 ± 0.02 ^a^	1.52 ± 0.02 ^a^	1.56 ± 0.03 ^a^	<0.001
16:0	13.89 ± 0.07 ^c^	12.54 ± 0.05 ^a^	13.08 ± 0.03 ^b^	13.34 ± 0.03 ^b^	<0.001
18:0	3.69 ± 0.02	3.66 ± 0.03	3.66 ± 0.02	3.58 ± 0.14	0.866
16:2n4	0.15 ± 0.01	0.11 ± 0.01	0.12 ± 0.01	0.12 ± 0.01	0.051
18:1n7	2.70 ± 0.01	2.49 ± 0.02	2.43 ± 0.05	2.23 ± 0.18	0.510
18:1n9	20.48 ± 0.13 ^a^	23.29 ± 0.01 ^b^	23.22 ± 0.33 ^b^	24.13 ± 0.35 ^b^	0.004
20:1n9	0.94 ± 0.01 ^c^	0.76 ± 0.01 ^a^	0.82 ± 0.01 ^b^	0.83 ± 0.01 ^b^	<0.001
18:2n6	21.80 ± 0.08 ^a^	31.13 ± 0.09 ^b^	30.89 ± 0.34 ^b^	30.83 ± 0.14 ^b^	<0.001
18:3n3	2.54 ± 0.02	3.50 ± 0.01	3.50 ± 0.04	3.00 ± 0.42	0.144
18:4n3	0.55 ± 0.01	0.49 ± 0.01	0.46 ± 0.02	0.45 ± 0.03	0.302
20:4n6, ARA	1.44 ± 0.05 ^b^	0.91 ± 0.03 ^a^	0.81 ± 0.15 ^a^	0.71 ± 0.03 ^a^	0.020
20:5n3, EPA	4.37 ± 0.04 ^b^	2.66 ± 0.02 ^a^	2.79 ± 0.21 ^a^	2.66 ± 0.02 ^a^	0.002
22:5n3	1.42 ± 0.02 ^a^	0.97 ± 0.01 ^b^	1.04 ± 0.03 ^b^	0.92 ± 0.11 ^b^	<0.001
22:6n3, DHA	11.64 ± 0.01 ^b^	6.79 ± 0.02 ^a^	6.73 ± 0.05 ^a^	6.70 ± 0.40 ^a^	<0.001
Other FA	5.88 ± 0.26	4.35 ± 0.01	4.33 ± 0.30	5.12 ± 1.11	0.468
ΣSFA	19.51 ± 0.10 ^c^	17.63 ± 0.09 ^a^	18.27 ± 0.07 ^b^	18.40 ± 0.02 ^b^	<0.001
ΣMUFA	27.99 ± 0.17	29.56 ± 0.03	29.51 ± 0.47	30.44 ± 0.08	0.610
ΣPUFA	45.89 ± 0.01	46.93 ± 0.03	46.35 ± 0.25	45.54 ± 1.09	0.176
Σn-3	22.65 ± 0.02 ^b^	15.89 ± 0.01 ^a^	15.69 ± 0.44 ^a^	14.07 ± 1.18 ^a^	0.005
Σn-6	23.24 ± 0.03 ^a^	32.04 ± 0.12 ^b^	31.66 ± 0.19 ^b^	31.47 ± 0.09 ^b^	<0.001
Σn-9	3.64 ± 0.02	3.26 ± 0.01	3.26 ± 0.08	3.02 ± 0.35	0.368
n3/n6	0.97 ± 0.01 ^b^	0.50 ± 0.01 ^a^	0.50 ± 0.02 ^a^	0.45 ± 0.04 ^a^	<0.001
EPA/DHA	0.38 ± 0.01	0.39 ± 0.01	0.41 ± 0.03	0.39 ± 0.01	0.691

CTF: fishmeal-based control diet containing 20% fishmeal and 9% fish oil; CTV: plant-based control diet containing 5% fishmeal and 5% fish oil; IB10: CTV supplemented 1% LB-IMMUNOboost; LP10: CTV supplemented 1% LB-LIVERprotect. Values are mean ± SEM (*n* = 9). Values in the same row with different lowercase letters indicate significant differences among dietary treatments (*p <* 0.05). EPA: Eicosapentaenoic acid; ARA: Arachidonic acid; DHA: Docosahexaenoic acid; Other FA: Other fatty acids; SFA: saturated fatty acids; MUFA monounsaturated fatty acids; PUFA: polyunsaturated fatty acids.

**Table 5:** Histomorphology analysis of the anterior intestine in juvenile *S. aurata* fed the diets.

	CTF	CTV	IB10	LP10	*p*-Value
FL (µm)	1526.8 ± 35.0 ^b^	1364.0 ± 35.1 ^ab^	1307.1 ± 38.0 ^ab^	1321.8 ± 27.4 ^a^	0.048
TEH (µm)	40.2 ± 0.6 ^b^	35.0 ± 0.6 ^a^	39.9 ± 1.0 ^b^	37.2 ± 0.7 ^ab^	<0.001
LPT (µm)	9.4 ± 0.3 ^a^	10.4 ± 0.4 ^ab^	10.6 ± 0.4 ^ab^	11.5 ± 0.4 ^b^	0.003
GC	2.7 ± 0.1 ^b^	2.7 ± 0.1 ^b^	2.0 ± 0.1 ^a^	2.6 ± 0.1 ^b^	0.002
EAA (µm^2^)	36.8 ± 0.8	30.26 ± 0.8	33.9 ± 0.6	35.9 ± 0.7	0.072

Fold length (FL); total enterocyte height (TEH); lamina propria thickness (LPT); number of goblet cells per 100 µm (GC); and enterocyte apical area (EAA). CTF: fishmeal-based control diet containing 20% fishmeal and 9% fish oil; CTV: plant-based control diet containing 5% fishmeal and 5% fish oil; IB10: CTV supplemented 1% LB-IMMUNOboost; LP10: CTV supplemented 1% LB-LIVERprotect. Values are mean ± SEM (*n* = 9). Values in the same row with different lowercase letters indicate significant differences among dietary groups (*p* < 0.05).

## Data Availability

The raw data supporting the conclusions of this article will be made available by the authors on request.

## Abbreviations

The following abbreviations are used in this manuscript:

EAA	Enterocyte apical area
FA	Fatty acid
FL	Intestinal fold length
GC	Number of goblet cells per 100 µm
HUFA:	Highly unsaturated fatty acids
LPT	Lamina propria thickness
MUFA	Monounsaturated fatty acids
PUFA	Polyunsaturated fatty acids
SFA	Saturated fatty acids
SEM	Scanning electron microscopy
TBARS	Thiobarbituric acid-reactive substances
TEH	Total enterocyte height
